# Post-craniopharyngioma surgery hypocalcemia due to denosumab use for osteoporosis: A case report

**DOI:** 10.1097/MD.0000000000039649

**Published:** 2024-09-13

**Authors:** Liangliang Jia, Yueqin Hu, Guilan Jin

**Affiliations:** a Department of Pharmacy, Yichang Central People’s Hospital, Yichang, Hubei, China; b Institute of Pharmaceutical Preparation, China Three Gorges University, Yichang, Hubei, China.

**Keywords:** adverse effects, dase reports, denosumab, hypocalcemia, osteoporosis

## Abstract

**Rationale:**

Denosumab, a fully humanized IgG monoclonal antibody, is commonly employed in the management of different types of osteoporosis. Up to now, hypocalcemia linked with denosumab has been predominantly reported in dialysis patients suffering from chronic kidney disease. Interestingly, there have been no reports of hypocalcemia following craniopharyngioma surgery with the use of denosumab.

**Patient concerns:**

A 65-year-old male received a subcutaneous injection of denosumab (60 mg) as a treatment for osteoporosis following the resection of a craniopharyngioma. Remarkably, the patient developed hypocalcemia within 4 days post-injection. However, 6 months subsequent to the initial treatment, the patient underwent another subcutaneous injection of desmuzumab and once again experienced hypocalcemia.

**Diagnoses:**

Hypocalcemia.

**Interventions:**

The hypocalcemia was successfully managed with intravenous calcium gluconate and oral calcium carbonate D3 tablets, leading to the alleviation of symptoms.

**Outcomes:**

Hypocalcemia following the use of denosumab after craniopharyngioma surgery is rare, and its occurrence may be associated with the primary disease and concomitant medications.

**Lessons:**

It underscores the necessity for clinicians to perform a thorough evaluation of the patient’s overall health status, complete all requisite testing, pay particular attention to those in high-risk categories, and ensure serum calcium levels are monitored, along with conducting other essential tests, prior to and following each administration of denosumab.

## 1. Introduction

Craniopharyngioma is a congenital benign intracranial tumor, accounting for 2% to 7.5% of intracranial tumors. Pituitary hypofunction occurs in 65% to 100% of patients after surgical treatment.^[1]^ Panhypopituitarism causes a series of hormone secretion deficiencies, leading to hypofunction of different glands and corresponding organ dysfunction. For example, hypothyroidism may be manifested by symptoms such as fear of cold, forgetfulness, apathy, pretibial myxedema, thin skin, sparse eyebrows, and hair. Additionally, amenorrhea and infertility may occur in patients with hypogonadism, while adrenal hypofunction can lead to anorexia, low blood pressure, or hypoglycemia.^[2]^ Hypopituitarism seriously affects the quality of life of patients and can even threaten their lives. Active and reasonable hormone replacement therapy can reduce mortality and improve the quality of life. However, long-term use of hormones, especially glucocorticoids, can cause a series of adverse reactions, such as glucocorticoid-induced osteoporosis (GIOP).^[3]^

Denosumab is a fully human IgG monoclonal antibody with high affinity and specificity for Receptor Activator of Nuclear Factor-**κ** B Ligand, which can block the combination of Receptor Activator of Nuclear Factor-**κ** B Ligand and Receptor Activator of Nuclear Factor-**κ** B, inhibit the activity and differentiation of osteoclasts, and thereby inhibit bone resorption.^[4]^ It is clinically used for the treatment of various types of osteoporosis. Some clinical evidence has been obtained for the treatment of GIOP with denosumab. A retrospective study analyzed 48 patients with GIOP, who were divided into bisphosphonate pretreatment group and bisphosphonate naive group. Bone mineral density and bone turnover markers were measured at baseline, 6 months, and 12 months. Denosumab significantly reduced bone turnover marker levels in both groups and increased bone mineral density at the lumbar spine and total hip compared to baseline.^[5]^ The most common adverse events associated with the use of denosumab were nonspecific musculoskeletal pain and limb pain, followed by a high risk of infection, hypocalcemia, skin-related disorders, and osteonecrosis of the jaw. At present, reports of hypocalcemia caused by denosumab mainly appear in dialysis patients with chronic kidney disease.^[6]^ Hypocalcemia after craniopharyngioma surgery has not been reported.

## 2. Case presentation

The patient was a 65-year-old man, underwent craniotomy for craniopharyngioma 3 years ago, and was diagnosed with “hypopituitarism” and “central diabetes insipidus,” for which he received hormone replacement therapy. The current treatment regimen includes desmopressin acetate 0.1 mg TID, testosterone undecanoate 40 mg TID, levothyroxine 87.5 µg QD, and hydrocortisone 20 mg (8 am) and 10 mg (4 pm). In the past year, the patient had experienced lower back pain without any significant external force. Dual-energy x-ray absorptiometry results show T scores for the lumbar spine as L1, −2.9; L2, −2.9; L3, −1.8; and L4, −2.1, diagnosed as “severe osteoporosis.” The patient was diagnosed with severe osteoporosis and was admitted to the hospital for osteoporosis treatment with denosumab..

**Figure 1.**
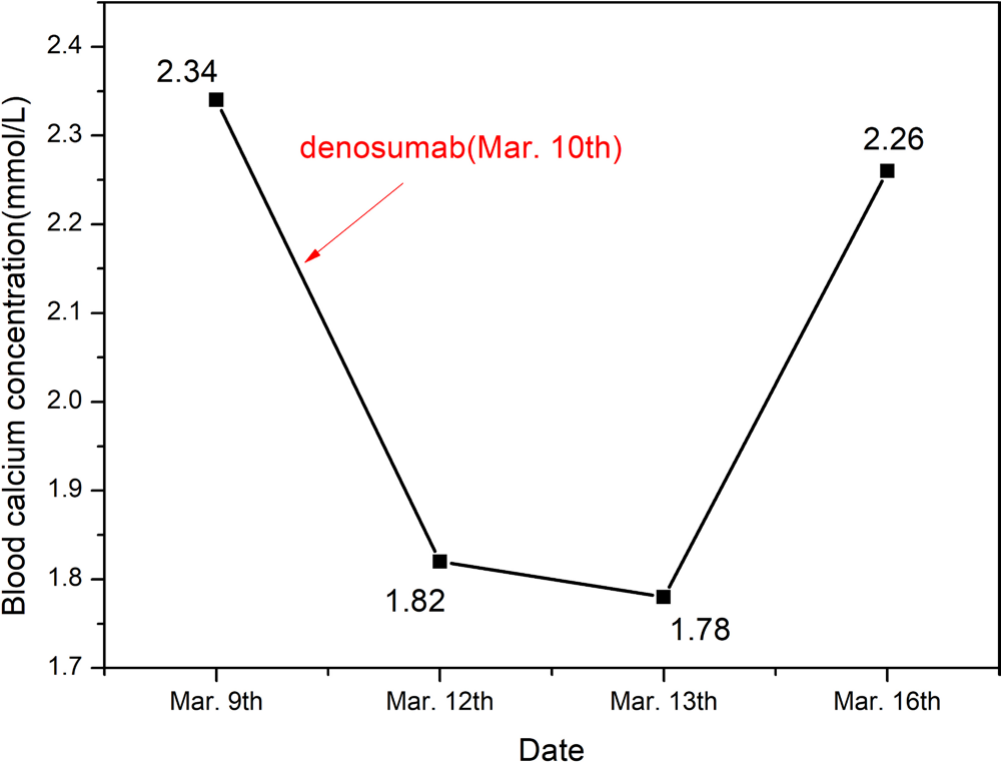
Changes in blood calcium concentration after the first administration of denosumab. Mar. = March.

Before treatment, the patient’s parathyroid hormone (PTH) level was 8.64 pg/mL, 25-hydroxyvitamin D level was 30 ng/mL, blood calcium concentration was 2.34 mmol/L, and blood phosphorus concentration was 1.22 mmol/L. After receiving a 60-mg subcutaneous injection of denosumab, the patient’s blood calcium concentration decreased to 1.82 mmol/L, and the blood phosphorus concentration was 0.52 mmol/L on the third day after administration. The doctor diagnosed hypocalcemia and immediately administered 1 g of calcium gluconate intravenously, along with oral calcium carbonate D3 chewable tablets at a dose of 0.75 g QD. Four days later, the patient’s blood calcium concentration increased to 2.26 mmol/L (Fig. 1), and the blood phosphorus concentration returned to the normal range (1.3 mmol/L). Consequently, the doctor deemed the hypocalcemia corrected and discharged the patient. After discharge, the patient’s medication regimen included 600 mg BID of calcium carbonate D3 chewable tablets and 0.5 µg QD of alfacalcidol soft capsules.

**Figure 2.**
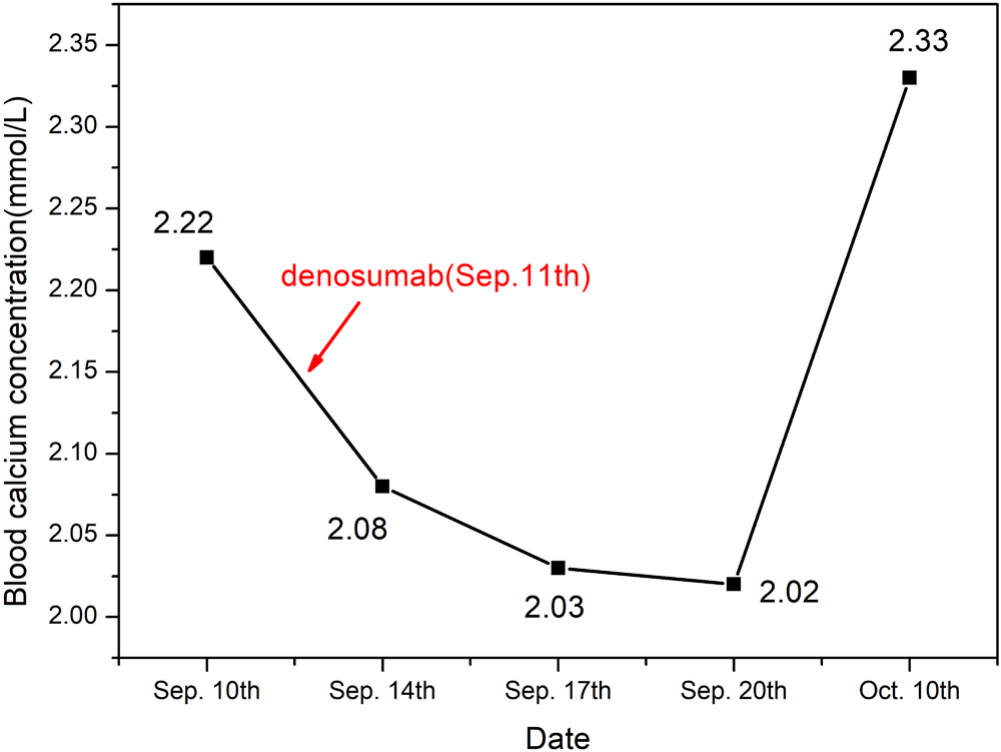
Changes in blood calcium concentration after the second administration of denosumab. Oct. = October, Sep. = September.

Six months after being discharged, the patient returned to the hospital for anti-osteoporosis treatment. Before receiving denosumab, the patient’s PTH level was 13.67 pg/mL, 25-hydroxyvitamin D level was 36 ng/mL, blood calcium concentration was 2.22 mmol/L, and blood phosphorus concentration was 1.19 mmol/L. On the 4th day after treatment, the patient’s blood calcium concentration decreased to 2.08 mmol/L (Fig. 2), and the blood phosphorus concentration was 1.18 mmol/L. The doctor administered a 1-g calcium gluconate injection intravenously and prescribed calcium carbonate D3 chewable tablets at a dose of 0.75 g QD. After 5 days of treatment, the patient’s blood calcium concentration remained around 2.0 mmol/L. Considering the blood calcium level to be relatively stable, the doctor instructed the patient to continue oral calcium supplementation after discharge. The treatment regimen included 0.5 µg QD alfacalcidol soft capsules and 0.6 g QD calcium carbonate tablets. Twenty days after discharge, the patient’s blood calcium concentration was rechecked and found to be 2.33 mmol/L.

## 3. Discussion

The occurrence of hypocalcemia after the use of denosumab is related to the primary disease, combined medication, and other factors. Specific risk factors for hypocalcemia include advanced tumor stage, low vitamin D levels, impaired renal function, and calcium malabsorption.^[7]^ Reduced 1-α-hydroxylase activity in patients with renal insufficiency can lead to impaired conversion of vitamin D to its active metabolite (1,25-hydroxyvitamin D), resulting in reduced intestinal absorption of calcium, and may be a contributing factor to hypocalcemia.^[8]^ For patients with secondary hyperparathyroidism, the parathyroid gland will be stimulated by hypocalcemia, hypomagnesemia, or hyperphosphatemia to secrete PTH under the action of the primary disease. If these patients are not treated in time, they will have a high risk of hypocalcemia, thus increasing the probability of denosumab-related hypocalcemia. Studies have shown that gastric cancer is an independent risk factor for hypocalcemia.^[9]^ For patients after gastrointestinal surgery, due to the combined effects of anatomical changes, postoperative dietary changes, and preoperative malnutrition, patients have micronutrient deficiencies after gastrointestinal surgery, and gastrointestinal surgery has been confirmed to lead to calcium and vitamin D absorption disorders.^[10]^ At the same time, vitamin deficiency caused by calcium malabsorption after gastrointestinal surgery can also be secondary to hypocalcemia.

Craniopharyngioma is the most common congenital tumor in the sellar region. Because of the aggressive growth characteristics of craniopharyngioma, preoperative tumor invasion and intraoperative traction often cause hypothalamus and pituitary injury, leading to most complications after operation.^[11]^ Among them, blood calcium disorder is one of the postoperative complications of patients with craniopharyngioma,^[12]^ and it is also the most harmful complication to patients. The forms of calcium metabolism disorder are very complex, such as hypercalcemia and hypocalcemia, and the forms of calcium disorder in the same patient are different in different periods after operation. A study monitored the blood calcium concentration of 101 patients after craniopharyngioma resection and found that 11 patients had hypocalcemia, with an incidence of 10.9%.^[13]^

The excision of a craniopharyngioma and the subsequent medication regimen can also affect blood calcium levels. The hypopituitarism that results from the removal of a craniopharyngioma significantly impacts a patient’s quality of life, necessitating hormone replacement therapy. According to the guidelines for adult hypopituitarism hormone replacement therapy in the United States,^[2]^ the treatment for panhypopituitarism includes glucocorticoids, vasopressin, thyroid hormone, and sex hormones.

Studies have shown that the use of glucocorticoids is an independent risk factor for hypocalcemia, which reduces blood calcium levels by inhibiting intestinal calcium absorption, vitamin D activity, and renal calcium reabsorption.^[14]^ Hormones can induce or aggravate peptic ulcer, and proton pump inhibitors (PPIs) are usually required to prevent hormone-induced gastrointestinal mucosal damage. PPIs inhibit the activity of the H+-K+-ATP enzyme, which in turn reduces the secretion of H+ and pepsin. Calcium carbonate is the most common form of calcium salts found in chyme. For calcium to be absorbed, it must dissociate from the chyme in the acidic environment of the stomach and duodenum. About 20% to 30% of calcium is absorbed in the small intestine. However, the potent and sustained acid-suppressing effect of PPIs disrupts the acidic environment of the stomach and the proximal part of the duodenum, preventing the ionization of calcium ions in the chyme and delaying their absorption.^[15]^ Studies have reported that in short-term trials, participants taking omeprazole had a reduction in calcium absorption from 9.1% to 3.5% compared to the control group, potentially leading to hypocalcemia.^[16]^ Due to reduced calcium absorption, hypocalcemia can lead to a feedback increase in the secretion of PTH and 1,25(OH)2D, which stimulates the formation and increases the activity of osteoclasts. Osteoclasts secrete various hydrolases and collagenases, producing large amounts of lactic and citric acids, which facilitate the dissolution of bone matrix and bone salts, leading to osteoporosis. This can result in osteoporotic fractures under external stress. Furthermore, long-term use of PPIs may also cause secondary hypergastrinemia, which can increase bone resorption through elevated secretion of PTH.^[17,18]^ In summary, the use of PPIs may affect bone health by interfering with calcium absorption and increasing bone resorption, ultimately potentially increasing the risk of osteoporosis and related fractures.^[19]^ In this study, the patient took hydrocortisone tablets 30 mg QD for a long time due to hypopituitarism and took large doses of esomeprazole during hospitalization due to the detection of *Helicobacter pylori* (+). The combination of the 2 drugs may be related to the occurrence of hypocalcemia.

In addition, some studies found that the blood calcium of patients who used iron for a long time was in the normal range before medication, and hypocalcemia occurred after the use of denosumab. It is suggested that the combined use of iron and denosumab may lead to hypocalcemia. It has been reported^[20]^ that iron overload (inappropriate use of iron) may lead to hypocalcemia. The underlying mechanism is not fully understood. It has also been reported^[21]^ that the use of high doses of furosemide after dehydration worsened the hypocalcemia caused by denosumab. Circulating diuretics may induce hypocalcemia by increasing renal calcium excretion. In addition, long-term use of anticonvulsant drugs may increase the degradation of vitamin D and lead to hypocalcemia. Estrogen can cause hypocalcemia by inhibiting osteoclast bone resorption. Glucocorticoids inhibit calcium absorption in the intestine and stimulate renal tubular calcium excretion, leading to hypocalcemia. The use of gadolinium contrast agents may also interfere with the colorimetric determination of calcium and lead to rapid and reversible hypocalcemia.^[22]^ The primary diseases/combined medications leading to hypocalcemia and their possible mechanisms are listed in Table 1. Therefore, it is suggested that patients who use iron, circulating diuretics, anticonvulsants, estrogen, glucocorticoids, gadolinium contrast agents, and other drugs should pay special attention to the occurrence of hypocalcemia when combined with denosumab.

**Table 1:** The primary diseases/combined medications leading to hypocalcemia and their possible mechanisms.

Primary disease/combined medication	Possible mechanisms leading to hypocalcemia
Renal insufficiency	The reduced activity of 1-α-hydroxylase results in impaired conversion to 1,25-dihydroxyvitamin D
Secondary hyperparathyroidism	Stimulated by low blood calcium, low blood magnesium, or high blood phosphorus, PTH is secreted compensatorily, increasing the risk of hypocalcemia
Gastric cancer/gastrointestinal surgery	Affects the gastrointestinal absorption of calcium and vitamin D
Craniopharyngioma	Tumor invasion and traction during surgery cause damage to the hypothalamus and pituitary gland
Glucocorticoid	Inhibits intestinal calcium absorption, reduces vitamin D activity, and inhibits renal calcium reabsorption
PPIs	Disrupts the acidic environment in the stomach and proximal duodenum, preventing calcium ions in the chyme from ionizing, thereby delaying their absorption
Loop diuretics	Increases renal calcium excretion
Anticonvulsant medications	Increases the degradation of vitamin D
Estrogen	Inhibits osteoclast-mediated bone resorption
Gadolinium-based contrast agents	Interferes with the colorimetric determination of calcium

PPI = proton pump inhibitor, PTH = parathyroid hormone.

A study by Zhao et al^[23]^ analyzed the occurrence time of hypocalcemia in 41 cases of patients treated with denosumab and found that the shortest occurrence time of hypocalcemia was 4 days after medication and the longest was 4 months. Among the patients, 21.05% developed hypocalcemia within 7 days and 87.47% developed it within 1 month. In this study, the patient showed a significant decrease in blood calcium concentration about 4 days after the administration of the drug, and special attention should be paid to the blood calcium level when administering the drug. Therefore, it is better for patients to monitor their blood calcium level regularly in the first half of the month after medication and pay attention to whether there are symptoms of hypocalcemia in the later period.

When the blood calcium level of the human body decreases, the excitability of nerve and muscle increases. Mild patients show numbness, pain, fatigue, and convulsion around the limbs and mouth. Severe patients can lead to convulsion, spasm of larynx, limbs, and bronchus, seizures, and mental symptoms such as restlessness, disturbance of consciousness, and hallucination.^[24]^ The main effect of hypocalcemia on the cardiovascular system is arrhythmia caused by conduction block, and the typical manifestation of the electrocardiogram is QT interval and ST-segment prolongation. The severity of hypocalcemia is not completely consistent with the degree of hypocalcemia but is related to the speed and duration of hypocalcemia. In this study, the patient had asymptomatic hypocalcemia twice. In addition to the blood calcium index, the patient also had hypophosphatemia. The reason may be that the lack of vitamin D limits the absorption of phosphorus in the intestine, and another reason may be that the PTH resistance induced by denosumab is compensated. PTH not only regulates calcium homeostasis but also regulates serum phosphorus. Elevated PTH reduces proximal tubular reabsorption and increases urinary phosphorus excretion,^[25]^ while osteoclasts do not release bone phosphorus, leading to hypophosphatemia.

In order to prevent the occurrence of hypocalcemia caused by denosumab, predose assessment, including baseline calcium, PTH, renal function, and vitamin D levels, should be performed prior to treatment, which will help identify higher risk groups. Calcium and vitamin D levels should be corrected prior to administration. Studies have shown that at least 1000 mg of elemental calcium should be supplemented daily prior to denosumab treatment. Patients with a baseline serum 25(OH)D concentration of 12 to 20 ng·mL^−1^ were supplemented with at least 800 IU of vitamin D per day, and patients with a baseline 25(OH)D concentration >20 ng·mL^−1^ were supplemented with at least 400 IU of vitamin D per day to prevent the occurrence of symptomatic hypocalcemia.^[26]^ In clinical treatment, most of the patients were treated with intravenous combined with oral calcium, and 10% calcium gluconate infusion was used for intravenous calcium supplementation. The reason may be that compared with calcium chloride, calcium gluconate rarely causes tissue necrosis during extravasation.^[27]^ Calcium carbonate, calcitriol, and cholecalciferol are mainly used in oral calcium supplementation. The reason may be that the drug can take effect directly without liver activation after entering the body, which is faster and does not need to be metabolized by the kidney, which is more advantageous for patients with poor renal function.

In conclusion, hypocalcemia caused by denosumab is mostly mild and asymptomatic; it can still lead to symptomatic severe and persistent damage and even life-threatening situations. With the increasing clinical application of denosumab, it is foreseeable that more patients with hypocalcemia will appear. Therefore, before the first use of denosumab, it is necessary to comprehensively assess the patient’s condition, improve various examinations, focus on high-risk groups, and do a good job in monitoring blood calcium and other necessary examinations after each medication.

## Author contributions

**Writing – original draft:** Liangliang Jia.

**Writing – review & editing:** Yueqin Hu.

**Data curation:** Guilan Jin.
